# Interaction between m6A methylation and noncoding RNA in glioma

**DOI:** 10.1038/s41420-022-01075-5

**Published:** 2022-06-10

**Authors:** Nairong Tao, Tongxuan Wen, Tong Li, Lan Luan, Hai Pan, Yong Wang

**Affiliations:** 1 Department of Neurosurgery, Central Hospital Affiliated to Shenyang Medical College, Shenyang, China; 2 Department of Pathology, Central Hospital Affiliated to Shenyang Medical College, Shenyang, China; 3 Department of Orthopedic Surgery, the First Affiliated Hospital, College of Medicine, Zhejiang University, Hangzhou, 310003 Zhejiang China; 4 Central Laboratory, Central Hospital Affiliated to Shenyang Medical College, Shenyang, Liaoning 110024 P.R. China

**Keywords:** Non-coding RNAs, Cancer

## Abstract

Glioma is considered to be the most common brain malignancy in the central nervous system. At present, the aetiology of glioma is not clear. Due to its rapidly growth and easily recurrence, the prognosis of patients with glioma is very poor. N6-methyladenosine (m6A) methylation is an internal reversible modification in most RNAs, including messenger RNAs (mRNAs) and noncoding RNAs (ncRNAs). Recent studies have shown that the m6A regulators are abnormal expressed, and are extensively involved in the progression of glioma by targeting ncRNAs. Moreover, as the most important epigenetic regulators, ncRNAs can also affect the function of m6A regulators in glioma. This review summarized the expression and function of certain common m6A regulators in glioma. Also, the current review sum up the mutual interactions between m6A regulators and ncRNAs in glioma.

## Facts

- Glioma always combines with a poor prognosis due to its invasive growth and recurrence.
- M6A RNA methylation is a dynamic and reversible modification process, and m6A is extensively involved in the occurrence and development of multiple malignancies.
- Growing evidence has indicated that m6A RNA methylation-modified ncRNAs play important regulatory roles in gliomagenesis and malignant progression.

## Open questions

- What is the role of m6A RNA methylation in glioma?
- What is the mechanism of ncRNAs regulated by m6A methylation in the development of glioma?
- How can we apply these research results to clinical practice and bring benefits to glioma patients?

## Introduction

Glioma arises from glial cells and neurons in the nervous system [1]. It is the most common primary malignant tumour in the brain [2]. The disease grows fast, relapses easily and has a high mortality rate [3]. At present, the aetiology of glioma is not clear, but genetic factors [4–6] and ionizing radiation [7, 8] are considered potential risk factors. At present, the clinical treatment of glioma is mainly surgery, radiotherapy and chemotherapy [9], but the recurrence rate of glioma is still high [10]. In recent years, with advances in molecular biology, increasing evidence has shown that m6A modification and ncRNAs are closely related to the occurrence and development of glioma [11].

N6-methyladenine (m6A) methylation was first reported in 1974 [12]. It is considered to be the most common internal modification of RNA molecules in organisms [13, 14], not only in mRNA but also in a variety of ncRNAs [15]. It mainly occurs on the RRACH [11, 16] sequence of purine bases and is mainly operated by “Writers”, “Erasers” and “Readers” [17, 18]. The modification process means that the sixth nitrogen atom on the RNA molecule is methylated under the catalysis of methyltransferase [19], then the methylation site is identified by a specific protein and further plays a regulatory role by activating upstream and downstream target genes or signal pathways. The methylation sites can be demethylated under the effct of demethylase [20, 21]. Therefore, m6A is a dynamic and reversible process [22]. Numerous studies have shown that m6A RNA methylation plays an important role in the malignant progression of various tumours, including glioma, and may become a target for cancer therapy and a potential biomarker for predicting prognosis [23, 24].

NcRNAs are a great kind of molecules that exert there regulatory effects at RNA level, and only a small portion of ncRNAs can be translated into proteins [25]. According to the length of nucleotides, ncRNAs are always divided into ncRNAs, miRNAs and circRNAs [26, 27]. In the current study, ncRNAs can interfere with a variety of target genes, participate in a variety of physiological and pathological links [28], and play an important role in glioma [27].

An in-depth study of m6A RNA methylation and ncRNAs has revealed that m6A methylation regulatory factors affect the occurrence and development of tumours, including gliomas, through their interaction with ncRNAs. This article reviews the interaction between m6A RNA methylation regulatory factors and ncRNAs on the biological function of glioma.

### Abnormal expression of m6A regulatory factors in glioma

**Fig. 1:**
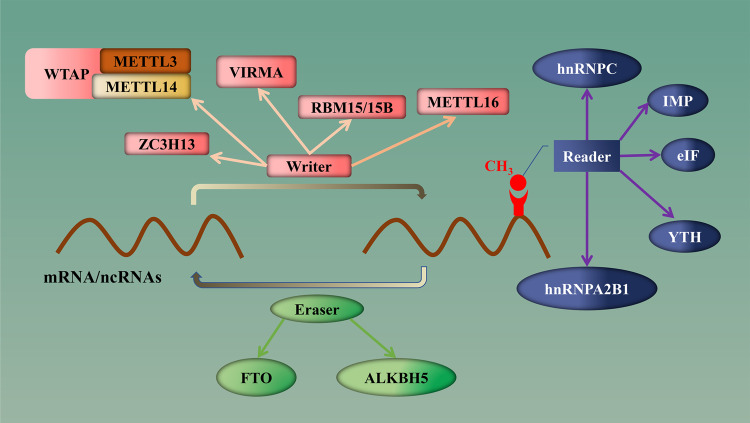
Classification diagram of m6A regulators. According to their regulatory functions, m6a regulators can be divided into Writers, Erasers and Readers. Writers (including METTL3, MTTTL14, WTAP, etc.) mainly play the role of methylation modification, while Erasers (including ALKBH5, FTO, etc.) mainly play the role of demethylation modification. Readers (including YTH family factors, IMP factors, eIF factors, etc.) mainly recognize methylated bases and play a regulatory role.

**Fig. 2:**
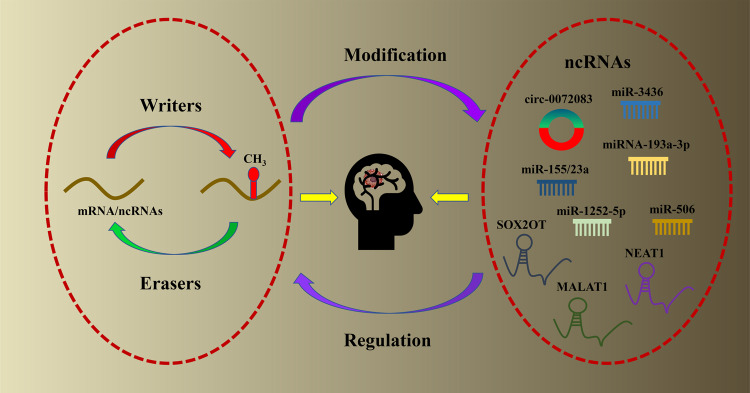
Mutual regulation between m6a regulator and ncRNAs in glioma. The interaction between m6a regulator and ncRNAs is briefly described, and the ncRNAs that play a role in glioma are listed, including lncRNAs, miRNAs and circRNAs.

At present, it has been found that there is abnormal expression of m6A methylation modification regulatory factors in a variety of tumours, which may be closely related to their occurrence and development [29]. In addition, the abnormal expression of m6A methylation modification regulatory factors has also been found in glioma [22, 30], in which “writers”, “erasers” and “readers” were involved in m6A methylation modification [16]. Simultaneously, m6A regulators are also considered to interact with ncRNAs and jointly affect the occurrence and development of glioma. [30] (Figs. 1 and 2).

#### Abnormal expression of writers in glioma

Methyltransferase, also known as a “writer”, catalyses the methylation of the sixth nitrogen atom in RNA [31]. At present, there are many known types of m6A methyltransferases, including methyltransferase-like 3 (METTL3), methyltransferase-like 14 (METTL14), tumour 1-associated protein (WTAP), virilizer like m6A methyltransferase-associated protein (VIRMA), RNA binding motif 15/15B (RBM15/15B), zinc finger CCCH domain-containing protein 13 (ZC3H13) and so on [32], which are involved in the core components of methyltransferases.

Wang and his colleagues analysed the expression of m6A regulators in gliomas and adjacent tissues in three databases and found 16 differentially expressed genes in gliomas and normal brain tissues. METTL3, METTL14, WTAP, RBM15, RBM15B, YTHDC2, YTHDF1, YTHDF2, YTHDF3, HNRNPA2B1 and HNRNPC levels were upregulated in gliomas, while ZC3H13 and FTO levels were downregulated [33].

In some studies, it has been found that the abnormal expression of methyltransferase is closely related to the occurrence and development of glioma. For example, Li and his colleagues examined METTL3 mRNA in cancerous and paracancerous tissues from 36 clinical patients with glioblastoma (GBM) and found that METTL3 was highly expressed in GBM tissues compared with normal brain tissues [34]. In addition, Cui et al. found that the differentiation ability of glioblastoma stem cells (GSCs) was significantly improved when the expression level of METTL3 was upregulated, and the growth and self-renewal of GSCs were significantly inhibited by this upregulation. In contrast, when the expression level of METTL3 or METTL14 in GSCs was downregulated, the growth and self-renewal ability of GSCs were significantly improved [35]. However, evidence from another group suggests that METTL3 has the opposite effect in GSCs. According to Visvanathan, METTL3 is highly expressed in GSCs, but its expression level decreases during GSC differentiation [36].

WTAP is reported to be highly expressed in glioma. By establishing a glioma cell silencing model, reducing the expression of WTAP can significantly inhibit the proliferation of glioma cells [37].

#### Eraser expression disorder in gliomas

Demethylases are also known as “erasers”. Fat mass and obesity-related protein (FTO), α-ketoglutarate-dependent dioxygenase alkB homologue 5 (ALKBH5), and α-ketoglutarate-dependent dioxygenase alkB homologue 3 (ALKBH3) are considered to be major demethylating enzymes [32]. Their main function is to remove the methyl group of the purine base of RNA molecules catalysed by methyltransferase [20].

Cui’s mouse experimental model showed that the lifespan of GSC-transplanted mice could be shortened by a decrease in FTO expression levels [35]. ALKBH5 has been found to be highly expressed in glioma and can eliminate the m6A methylation of G6PD through ALKBH5, improving the proliferation ability of glioma cells [38].

#### Abnormally expressed readers in glioma

Reader proteins, also known as “readers,” perform specific biological functions by specifically recognizing and binding RNA bases [20]. Current reader proteins include eukaryotic translation initiation factors (eIF3a, eIF3b, eIF3h), YTH n6-methyladenine sodium-binding proteins 1,2,3 (YTHDF1, YTHDF2, YTHDF3), YTH domain-containing proteins 1,2,3 (YTHDC1, YTHDC2, YTHDC3), insulin-like growth factor 2 mRNA binding protein 1,2,3 (IGF2BP1/IMP1, IGF2BP2/IMP2), heteronuclear protein A2B1 (hnRNPA2B1) and heteronuclear protein (hnRNPC) [32].

Previous studies have shown that YTHDF1 [39], YTHDF2 [39], IMP2 [40, 41] and hnRNPA2B1 [42] are highly expressed in gliomas, while hnRNPC [39] is poorly expressed in gliomas.

In the study and analysis of the expression of YTHDF2 in glioma and its mechanism, Feng et al. found that YTHDF2 was upregulated in glioma, and this high expression affected the proliferation, invasion and tumorigenesis of GBM cells. They found that this effect was mainly achieved by downregulating the expression of LXRa and HIVEP2. These effects were related to the poor prognosis of patients [43].

To investigate the role of hnRNP A2/B1 in the development and progression of gliomas, Deng and his colleagues systematically detected and analysed the expression levels of hnRNP A2/B1 in 40 glioblastoma tissues and normal brain tissues. They found that hnRNP A1/B1 was highly expressed in glioblastoma compared with normal brain tissues. When they downregulated the expression of hnRNPA2/B1 in glioblastoma, they found that the invasion, growth and viability of tumour cells were significantly reduced [44].

### Biological functions of m6A modification in glioma

M6A modification affects all aspects of the development and progression of gliomas, and its potential influencing factors may include invasion, proliferation, apoptosis, drug resistance, and tumorigenesis.

#### m6A regulators participate in the regulation of glioma proliferation

The m6A regulatory factor may be involved in influencing the proliferation of glioma cells. In Visvanathan’s experiments, METTL3 improved the stability of GSCs by increasing the m6A modification of SOX2 mRNA at the SOX2 site. In addition, in a mouse model, they also found that the expression level of METTL3 in mouse GBM tumours was also increased and reducing the expression level of METTL3 could reduce the growth ability of tumour cells, thereby prolonging the survival time of mice [36]. In another clinical study, Li and colleagues selected clinical tissue samples of GBM at different stages for experimental study. They found that the expression level of METTL3 in GBM tissues was significantly increased and may be closely related to the grade and poor clinical prognosis of GBM. To test this idea, they downregulated the expression of METTL3 in the GBM cell lines U87 and U251 and found that the invasion and tumorigenesis of GBM cells were significantly inhibited. Furthermore, they found that METTL3 also regulated alternative splicing of BCLX and NCOR2, promoting proliferation and self-renewal of GBM cells [34].

In one study, Yarmishyn and colleagues used the Oncomine database to analyse the expression levels of YTH family proteins in GBM, and they found that YTHDF1 had the highest expression levels in GBM. When the expression level of YTHDF1 in GBM cells was downregulated, the proliferation ability of GBM cells was decreased. In addition, they found that Musashi-1 (MSI1), a regulator associated with high carcinogenicity of GBM, could directly regulate the expression of YTHDF1, thereby affecting the proliferative capacity of GBM cells [45].

#### Effects of m6A regulators on glioma migration and invasion

Various studies have shown that the abnormal expression of regulatory factors involved in m6A modification can lead to changes in the migration and invasion of glioma cells.

An experimental study found that the expression of IMP2 was significantly increased in GBM, and further studies showed that IMP2 could regulate the PI3K/Akt signalling pathway by regulating IGF2 activity and promote the proliferation and invasion of GBM cells [40].

In an experimental study to verify the effect of METTL3 on the invasion and migration of glioma cells, Li et al. knocked down the expression level of METTL3 in U87 and U251 cells and found that the migration and invasion of U87 and U251 cells were significantly inhibited [34].

#### m6A regulators of apoptosis in glioma

M6A methylation can regulate the apoptosis of glioma cells, and Liang’s study showed that eIF3B was upregulated in the glioblastoma cell line U87. When eIF3B expression was downregulated in U87 cells by transfection, the proliferation of U87 cells was further reduced, and they noted that this reduction in proliferation was associated with an increase in apoptosis, suggesting that eIF3B also plays a key role in GBM cell apoptosis [46].

Yin and colleagues found that hnRNPA2/B1 is highly expressed in gliomas. Downregulation of hnRNP A2/B1 expression in the glioma cell line U251 further induced apoptosis of tumour cells. They found that the regulation of proliferation and apoptosis of glioma cells was mainly achieved by regulating AKT and TAT3 signalling pathways [47].

#### m6A methylation regulators affect glioma activity and tumorigenesis

It has been reported that r-2-hydroxyglutarate (r-2HG) is a tumour metabolite produced by the mutation of isocitrate dehydrogenase 1/2 (IDH1/2), which can increase the level of FTO m6A and inhibit tumour growth [48] and has antitumor effects. R-2HG also showed corresponding antitumor activity in glioma. Su and his colleagues also found that when R-2HG targeted the FTO/m6A/MYC/CEBPA signalling pathway, the activity of FTO was further reduced, and the proliferation and survival of tumour cells were inhibited, thus achieving antitumor effects [49].

In addition, as GSCs have the ability to promote tumour growth and invasion, they may be associated with poor prognosis in GBM patients. At the same time, they can also be regulated by m6A RNA methylation, and related studies have shown that m6A RNA methylation is related to GSC self-renewal and tumorigenesis [46].

#### Correlation between m6A methylation regulators and drug resistance in gliomas

One study revealed a correlation between m6A modification and glioma drug resistance. In this study,Ding and colleagues recruited 36 temozolomide-resistant patients and 33 temozolomide-sensitive patients and found that circ-0072083 was increased in temozolomide-resistant glioma tissues and cells and that decreased expression of circ-0072083 blocked demethylation of ALKBH5, thereby reducing NANOG expression. In addition,circ-0072083 can target miR-1252-5p to further regulate the expression of NANOG and ALKBH5, thereby controlling TMZ resistance. The expression level of circ-0072083 is abnormal, which has certain value in evaluating the prognosis of patients [50].

### Mutual regulation between m6A RNA methylation regulators and ncRNAs in glioma

**Fig. 3:**
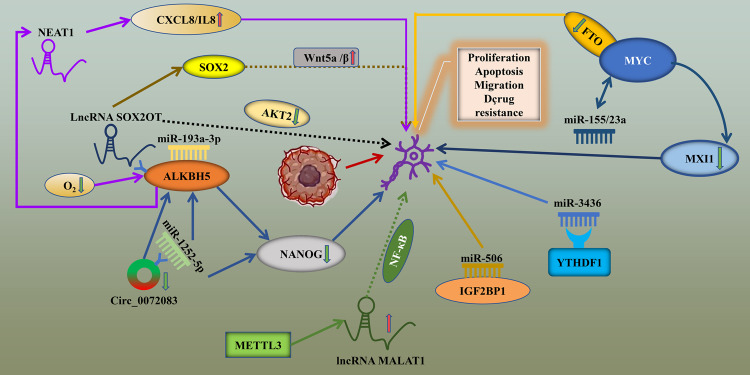
Schematic diagram of mutual targeting regulation of m6a regulators and ncRNAs in glioma. m6A regulators can interact with ncRNAs and affect the biological behavior of glioma cells, such as proliferation and invasion.

NcRNAs are one of the few RNA molecules that with limited translation ability transcripts, and most of them play a regulatory role at the transcriptional level [25]. ncRNAs mainly include miRNAs, lncRNAs and circRNAs [51]. Recent studies have shown that the regulatory factors of m6A RNA methylation modification in glioma can target ncRNAs to play a role in promoting or inhibiting cancer (Table 1). On the other side, ncRNAs can also affect the function of m6A regulators in glioma (Table 2). As demonstrated in Fig. 3 and illustrated accordingly, the mutual interactions between m6A regulators and ncRNAs are closely implicated in the progression of glioma.

**Table 1:** Role of m6A regulators targeting ncRNAs in glioma.

m6A regulators	Target ncRNAs	Functional classification	Mechanism	PMID
FTO	miRNA- 155/23a	Enhance the inhibitory effect of temozolomide on the proliferation of glioma cell line U87	FTO can target MYC-miRNA-MXI1 feedback loop and enhance the efficacy of TMZ by inhibiting FTO	32680921
METTL3	LncRNA MALAT1	Promote the malignant progression of IDH wild type glioma	METTL3 enhances the stability of MALAT1 through m6A modification, activates NF-κB pathway after up-regulation of MALAT1 expression, and promotes malignant progression of IDH wild-type glioma	33933553
ALKBH5	LncRNA NEAT1	Promote the malignant progression of GBM	Hypoxia induced ALKBH5 can also eliminate the expression of m6A in LncRNA NEAT1, increase the stability of transcripts and up regulate the expression of CXCL8 / IL8, so as to promote the progress of GBM	34670781

**Table 2:** Targeted regulation of m6A regulators by ncRNAs in glioma.

ncRNAs	Target m6A regulators	Functional classification	Mechanism	PMID
miRNA- 193a-3p	ALKBH5	Promote apoptosis of glioma cell lines U87 and U251	Cell experiments and mouse models show that miR-193a-3p can directly target ALKBH5 and further promote the apoptosis of tumour cells by inhibiting miR-193a-3p/ALKBH5/AKT2 signaling pathway in vivo and in vitro	33968717
miRNA-3436	YTHDF1	regulate the proliferation of glioma cells and is related to the poor prognosis of patients	miR-3436 can negatively regulate the expression of YTHDF1, regulate the proliferation of glioma cells, and is related to the poor prognosis of glioma patients	32449290
miRNA-506	IGF2BP1	Inhibit the growth and invasion of GBM cells	miR-506 can directly target and downregulate IGF2BP1 gene, which can inhibit the growth and invasion of glioma cells	26692944
LncRNA SOX2OT	ALKBH5	Promote GBM cell proliferation and TMZ resistance, inhibit GBM cell apoptosis and promote GBM malignant progression	LncRNA SOX2OT can target ALKBH5, bind its downstream SOX2 site to ALKBH5, increase the expression level of SOX2 through the demethylation of ALKBH5, and activate Wnt5a/β-Catenin signaling pathway promotes the malignant progression of tumour	32439916
LncRNA JPX	FTO	Promote aerobic glycolysis and TMZ chemotherapy resistance in GBM	LncRNA JPX can promote aerobic glycolysis and TMZ chemotherapy resistance in GBM in a m6A dependent manner by regulating FTO / PDK1 axis	34390075
circ-0072083	ALKBH5	Regulating glioma TMZ drug resistance	Down regulation of circ-0072083 can eliminate the demethylation modification of ALKBH5 and downregulate the expression level of NANOG to regulate glioma drug resistance	33975615

#### Role of m6A regulators targeting ncRNAs in glioma

Xiao’s study found that the high expression of miR-155 and miR-23a can not only promote glioma but also improve the proliferation of U87 glioma cells. In their study, they found a new feedback pathway that can regulate glioma cell proliferation and tumorigenesis, the MYC-miRNA-MXI1 pathway. In this pathway, MYC inhibits the expression level of MXI1 by affecting the expression of miR-155 and miR-23a, and MXI1 can also inhibit the expression level of MYC, thus forming a feedback loop to regulate glioma tumorigenesis and cell proliferation. They also found that the MYC feedback pathway can be targeted by FTO and improve the efficacy of temozolomide chemotherapeutic drugs and inhibit the proliferation of glioma cells by inhibiting the expression of FTO [52].

Furthermore, Chang and colleagues found that METTL3 was highly expressed in IDH wild-type gliomas, and the high expression of METTL3 was positively correlated with higher tumour malignancy and poor prognosis. They also further confirmed through in vivo and in vitro experiments that METTL3 can enhance the stability of MALAT1 through m6A modification and increase the expression level of MALAT1, which can activate NF-κB. This leads to the malignant progression of IDH wild-type glioma [53].

Studies have indicated that ALKBH5 is related to the genetic characteristics of hypoxia in patients with GBM. Hypoxia-induced ALKBH5 can also eliminate the expression of m6A in lncRNA NEAT1, increase the stability of transcripts and upregulate the expression of CXCL8/IL8 to promote the progression of GBM [54].

#### Effect of m6A regulators regulated by ncRNAs in glioma

To verify the expression and mechanism of miR-193a-3p in glioma, Cui and colleagues found that the expression level of miR-193a-3p decreased in glioma tissues and cell lines U87 and U251. Upregulating the expression level of miR-193a-3p in U87 and U251 cells promoted the apoptosis of tumour cells. Furthermore, their experiments in a xenograft mouse model also showed the same results. Moreover, they also found that miR-193a-3p can directly target ALKBH5 and further promote the apoptosis of tumour cells by inhibiting the mir-193a-3p/ALKBH5/AKT2 signalling pathway in vivo and in vitro [55].

In one study, Xu and his colleagues analysed the abnormal expression of the m6A regulatory factor in gliomas and screened the YTHDF1 factor according to its impact on the survival and prognosis of gliomas. In further experimental studies, they found that the expression of YTHDF1 was significantly upregulated in U87 and SHG-44 cells. Subsequently, they constructed a YTHDF1 inhibition model and overexpression model in U87 and SHG-44 cell lines, respectively, and investigated cell proliferation. The results showed that overexpression of YTHDF1 could significantly promote the proliferation of tumour cells. In addition, they also found that miR-3436 can negatively regulate YTHDF1, which is related to the poor prognosis of glioma patients [56].

Luo and colleagues found that miR-506 expression levels were significantly downregulated in GBM tissues and cell lines, and when miR-506 expression levels were upregulated, they could inhibit the growth and invasion of GBM cells. In addition, they also found that miR-506 can directly target the IGF2BP1 gene and can play a role in cancer suppression by regulating the expression level of IGF2BP1 [57].

Studies have shown that the lncRNA SOX2OT is upregulated in temozolomide-resistant and relapsed GBM tissue samples, and its high expression is considered to be closely related to GBM recurrence and poor prognosis. Silencing of lncRNA SOX2OT increased the sensitivity of GBM to temozolomide, increased apoptosis and decreased proliferation. They further studied its mechanism of action and revealed that lncRNA SOX2OT could target ALKBH5, bind the SOX2 site downstream of lncRNA SOX2OT to ALKBH5, and increase the expression level of SOX2 through the demethylation of ALKBH5. This activated the Wnt5a/β-catenin signalling pathway and promoted the malignant progression of tumours [58].

When investigating the relationship between lncRNA JPX and TMZ resistance in GBM, the researchers found that JPX was highly expressed in GBM tissues and cell lines and believed that this had important implications for the poor prognosis of patients. Through further experimental studies, they found that silencing lncRNA JPX could significantly interact with FTO and PDK1 mRNA and could promote aerobic glycolysis and resistance to TMZ chemotherapy in GBM in a m6A-dependent manner by regulating the FTO/PDK1 axis [59].

Circ-0072083 is present in exosomes of drug-resistant cells, and some studies have found that it is highly expressed in temozolomide-resistant glioma tissues and cells. When circ-0072083 is downregulated, the demethylation of ALKBH5 is eliminated, and the expression level of NANOG is downregulated, thus achieving the regulation of drug resistance in glioma. In addition, circ-0072083 can also target miR-1252-5p to affect the expression of NANOG and ALKBH5 and regulate the drug resistance of glioma [50].

## Conclusion

Glioma is the most common type of tumour in the central nervous system, with a high mortality rate. Currently, the main clinical treatment for glioma is the integration of various therapies, including surgery, radiation and chemotherapy. Because of the high recurrence rate, the prognosis of patients is still poor. In recent years, with the in-depth study of molecular biology, m6A has been found to be closely related to the occurrence and development of a variety of tumours. At the same time, the regulation of ncRNAs by m6A methylation is also considered to affect the malignant progression of many tumours. m6A methylation has become a potential biomarker for targeted therapy and prognosis prediction of tumours, while its roles and functions in gliomas remain unclear. Further study on the role and function of m6A methylation-regulated noncoding RNAs in glioma will provide new ideas for the clinical targeted therapy of glioma in the future.

## Supplementary information

language certification

## Data Availability

All data generated or analyzed in this study are included in this published article.
